# Morphology of subsurface cracks in glass-ceramics induced by Vickers indentation observed by synchrotron X-ray multiscale tomography

**DOI:** 10.1038/s41598-022-11084-0

**Published:** 2022-04-28

**Authors:** Gaku Okuma, Kei Maeda, Satoshi Yoshida, Akihisa Takeuchi, Fumihiro Wakai

**Affiliations:** 1 Research Center for Structural Materials, National Institute for Materials Science, 1-2-1 Sengen, Tsukuba, Ibaraki 305-0047 Japan; 2 Department of Materials Science and Technology, Tokyo University of Science, 6-3-1 Niijuku, Katsushika-Ku, Tokyo, 125-8585 Japan; 3 AGC Inc. Yokohama Technical Center, 1-1 Suehiro-cho, Tsurumi-ku, Yokohama, 230-0045 Japan; 4 Japan Synchrotron Radiation Research Institute, JASRI/SPring-8, Kouto 1-1-1, Sayo, Hyogo 679-5198 Japan

**Keywords:** Materials science, Structural materials, Ceramics, Glasses

## Abstract

The characterization of subsurface cracks induced by indentation is a challenge for understanding contact damage, impact, wear, erosion, and abrasion of brittle materials, because the crack pattern observable on the surface is only a part of the total crack system. Here we applied synchrotron X-ray multiscale tomography to observe the morphology of subsurface cracks produced by Vickers indentation in a novel CaO–Al_2_O_3_–SiO_2_ glass-ceramic with plate-like crystals forming a house-of-cards microstructure. It revealed a diverse type of crack systems around the semispherical microcrack zone beneath the indent, including a new mode II inclined lateral crack driven by the maximum shear stress. Tomography images provided knowledge on how the heterogeneous microstructure affected the toughening processes such as crack deflection, crack bridging, and microcracking.

## Introduction

Crack patterns on the surface induced by sharp or blunt indenter provide rich knowledge on mechanical properties of brittle materials, such as glass, glass-ceramic, ceramics, and composites, for developing damage resistant materials in structural, dental and optical applications. Vickers indenter generates radial^1,2^, median^1,2^, and lateral cracks^1,3^, which initiate at the boundary of elastic–plastic deformation zone, or, process zone. The driving force of crack formation is the maximum principal stress around the elastic–plastic deformation zone^4^; $\sigma_{\phi \phi }$ on the surface ($\theta = \pi /2$) for radial crack, $\sigma_{\theta \theta }$ at the bottom of process zone ($\theta = 0$) for median crack, and $\sigma_{rr}$ ($\theta = 0$) for lateral crack, where $\theta$ is the angle to the loading axis in spherical polar coordinates, $\phi$ is the hoop angle about the loading axis, and *r* is the radial distance. The initiation and growth of cracks in transparent materials are observed directly by optical microscopy under loading and unloading sequences^5^. The crack pattern depends on the local deformation behavior in the process zone, for example, densification and shear flow in glass^6,7^. The 3D crack system will be more complex in tough materials having heterogeneous microstructure with weak internal interfaces and high internal residual stress. Spherical indenter leads to Hertzian cone crack or subsurface deformation zone beneath the contact^8^. This microcrack damage zone is associated with nonlinear stress–strain curves, or quasi-plasticity^9^, in mica glass-ceramic^10^ and heterogeneous ceramics.

The complicated subsurface indentation crack system has been studied by observing the cross-section using optical microscopy^8,11,12^ and scanning electron microscopy (SEM)^13,14^. Focused ion beam (FIB) tomography can be used as a serial sectioning technique^15,16^. However, these sectioning methods affect the stress field around the process zone, so that it may alter the original morphology of crack system. X-ray computed tomography (CT) is a powerful technique to observe the internal cracks non-destructively^17^. Lacondemine et al.^18^ performed in-situ Vickers indentation experiment by means of X-ray tomography, and assessed the displacement field using a Digital Volume Correlation routine (DVC). Okuma^19^ clearly detected crack-like defects formed during powder processing and sintering of alumina by using a multiscale X-ray computed tomography, which was developed by Takeuchi and co-workers in SPring-8^20,21^.

The purpose of this work is to investigate the complicated 3D crack system generated in brittle materials with heterogeneous microstructure by using the multiscale X-ray computed tomography. Here, we used a translucent glass-ceramic as a model material. The glass-ceramics are defined as inorganic, non-metallic materials prepared by controlled crystallization of glasses via different processing methods^22,23^. A wide variety of glass-ceramic with heterogeneous microstructures have been developed by controlling the chemical composition of glass and the size, shape, and volume fraction of crystalline phase embedded in the glass for improving strength and fracture toughness^24–26^. The residual stresses that arise from thermal expansion and elastic mismatch between the crystal and the glass affect the mechanical properties of glass-ceramic^27^. The possible toughening mechanisms applicable to glass-ceramic are crack bowing^28^, crack deflection^28,29^, crack bridging^30,31^, and microcrack toughening^32,33^. A novel glass-ceramic composed of a CaO–Al_2_O_3_–SiO_2_ glass and hexagonal CaAl_2_Si_2_O_8_ crystals (h-CAS) was found by Maeda^34,35^ recently. This glass-ceramic (CAS-GC) exhibited improved fracture toughness and non-linear load–displacement curves in bending tests using the Single Edge V-Notched Beam (SEVNB) specimens^34,36^. The crack propagation is influenced by a house-of-cards microstructure formed by plate-like h-CAS crystals^36,37^. Cracks travel along the glass-crystal interface and the cleavage plane, since the h-CAS crystal structure is analogous to that of mica^38^. The CAS-GC is highly resistant to the abrasion damage^39^. We investigated 3D crack structure induced by Vickers indentation in CAS-GC by using the multiscale X-ray computed tomography. An attempt was made to understand the complicated subsurface crack system as a collection of crack components.

## Results

### Indentation cracking observed by optical microscopy

**Figure 1:**
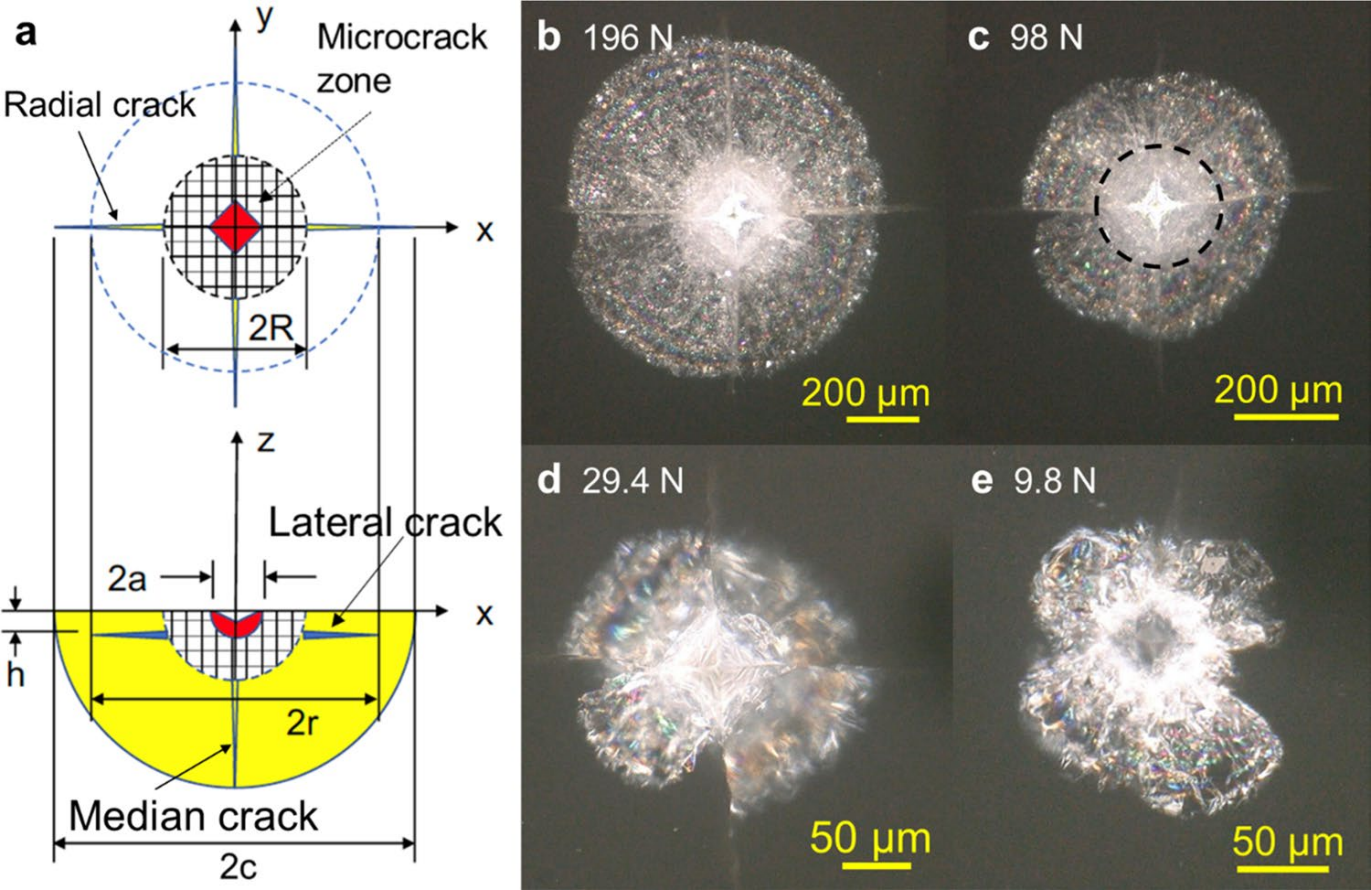
Optical micrograph of cracks induced by Vickers indentation in CaO–Al_2_O_3_–SiO_2_ glass-ceramic (CAS-GC). (**a**) An idealized crack structure, (**b**) indentation load 196 N, (**c**) 98 N, (**d**) 29.4 N, (**e**) 9.8 N.

Figure 1 shows Vickers indentation crack patterns for various loads in translucent CAS-GC observed by optical microscopy. The indentation cracks are symmetric at high loads, 196 N and 98 N, as schematically illustrated in Fig. 1a. The subsurface lateral crack is seen as a brilliant circular area (Fig. 1b,c), in contrast to smooth lateral cracks observed in glasses (Supplementary Fig. S1). Four radial cracks on the surface emanate from corners of the indent. The microcrack zone is defined as a circular opaque white area at the center (dashed line in Fig. 1c). The lateral crack system is composed of four circular sectors, the shape and size of which are irregular at loads less than or equal to 29.4 N (Fig. 1d,e). The radii of radial crack *c*, lateral crack *r*, microcrack zone *R*, and indent size *a* increase with indentation load *P* according to power law relationship

1 $$L \propto P^{n}$$

where *L* represents the radii and *n* is an exponent (Supplementary Fig. S2). The exponents for radial crack and lateral crack were 0.57 and 0.65, respectively. They were approximately equal to the theoretical value of 2/3 for radial crack^2^ and 5/8 for lateral crack^3^. The exponent of 0.73 for microcrack zone was closer to the value of 0.57 for radial and 0.66 for lateral crack systems than the value of 0.5 for indent size.

### Micro-CT observation of Vickers indentation cracks at 29.4 N load

**Figure 2:**
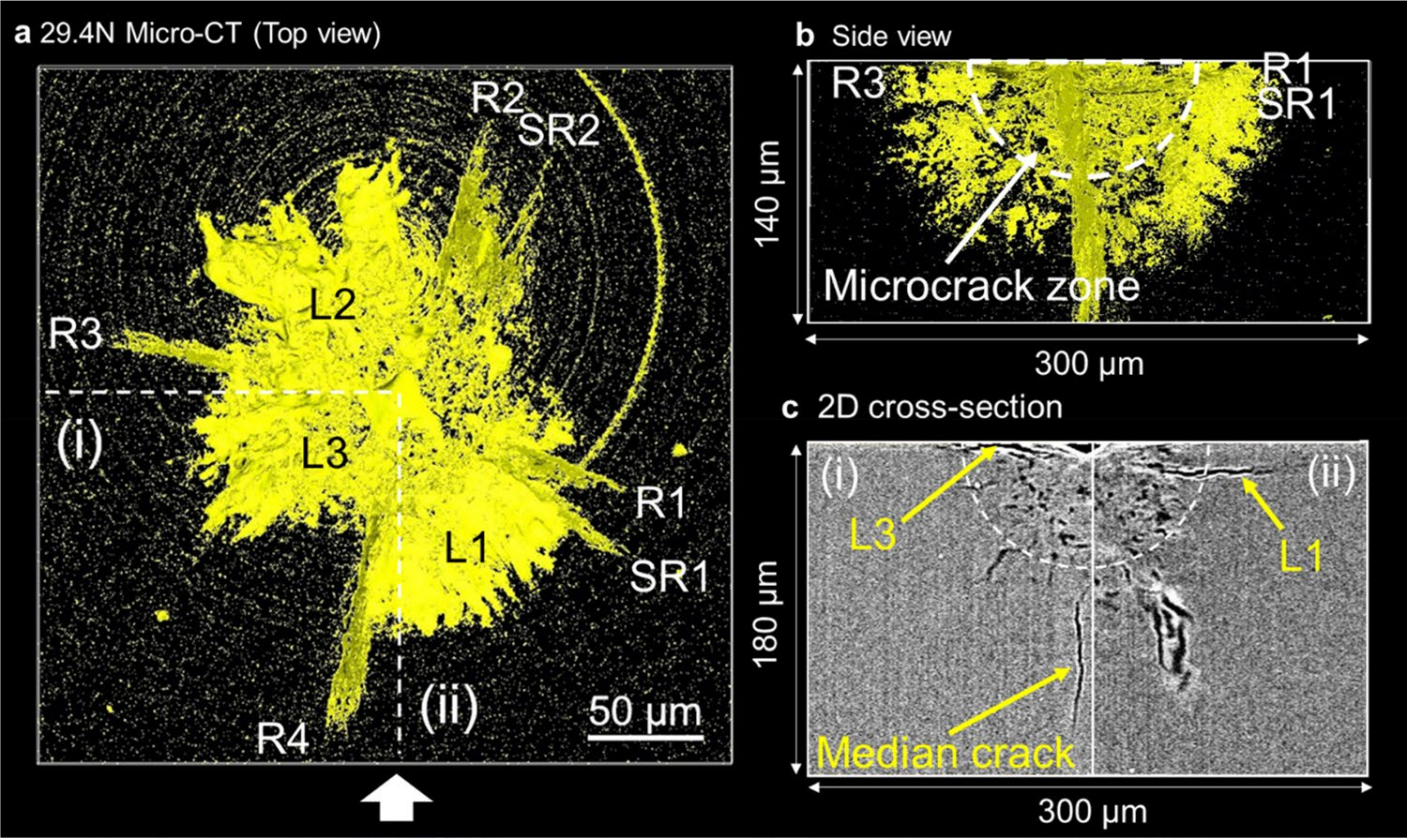
Micro-CT observation of Vickers indentation cracks at 29.4 N load. (**a**) Top view, (**b**) side view, (**c**) 2D cross-sectional image along white dashed lines (i) and (ii) in (**a**).

Figure 2a represents the top view of micro-CT image of a CAS-GC specimen indented at 29.4 N load (see also Supplementary Movie 1). Ring artifacts appear as circles centered on the axis of rotation of the sample during data acquisition. There were four primary radial cracks (R1–R4), two of them accompanying secondary radial cracks (SR1, SR2) which emanated adjacent to indentation corners. Three asymmetric shallow lateral cracks (L1, L2, L3) had irregular shapes. The shallow lateral crack (L1) was bounded by a secondary radial crack (SR1). The secondary radial crack (SR2) inclined to the loading axis. The side view in Fig. 2b showed the semispherical microcrack zone. The radial/median crack system looked like a so-called half-penny crack with annular geometry, because both radial crack and median crack initiated near the boundary of the microcrack zone and extended outward. However, radial cracks R1, SR1, R3 were not actually connected in the region below the microcrack zone. The cross-sections along lines (i) and (ii) in Fig. 2a are illustrated in Fig. 2c, indicating shallow lateral cracks (L1 and L3) parallel to the surface and a median crack perpendicular to it. This median crack was connected with radial crack R4. The microcrack zone or the damage zone in the semicircle (dashed line in Fig. 2c) contained numerous small dark spots, which corresponded microcracks. The shallow lateral cracks initiated inside the microcrack zone.

**Figure 3:**
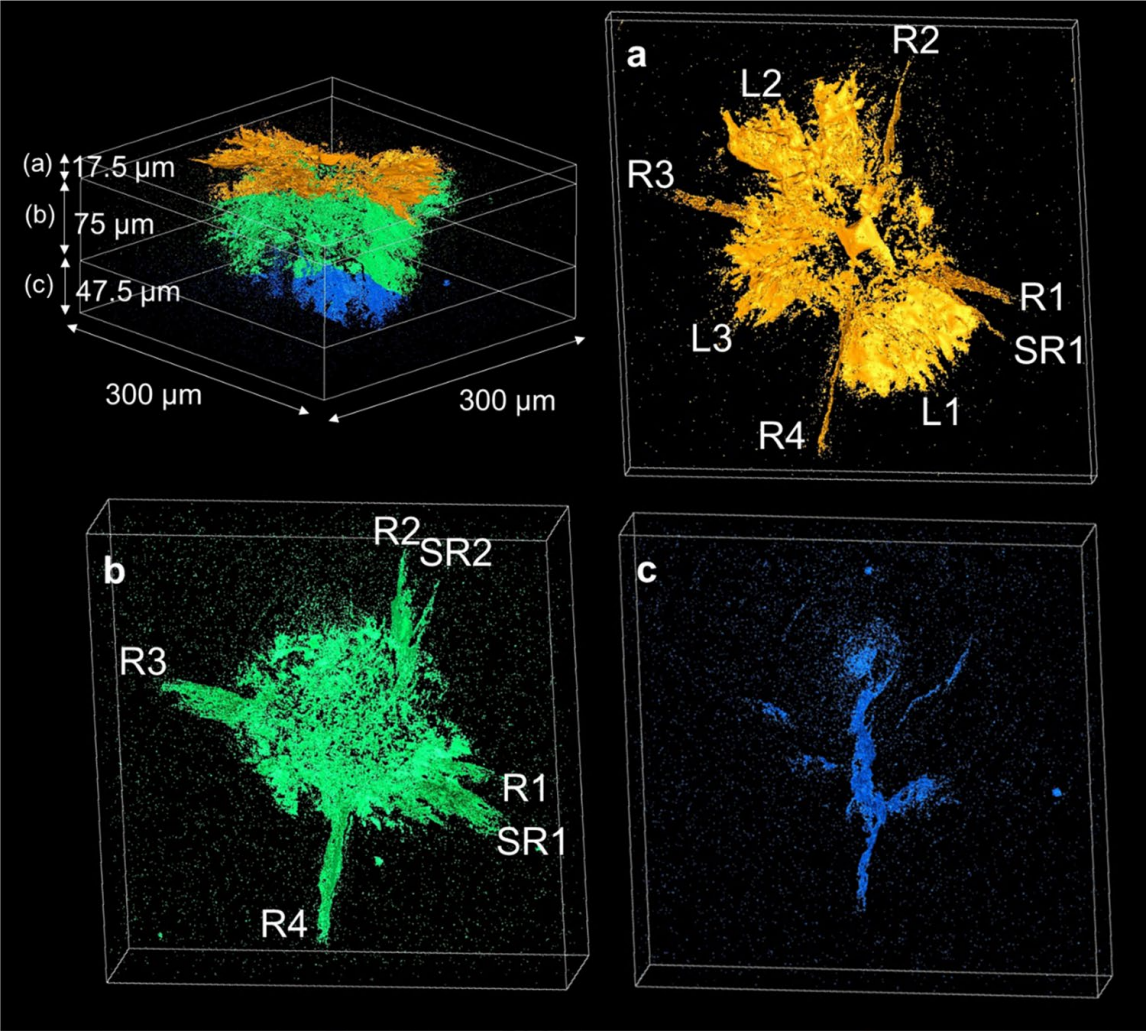
Layer structure of subsurface cracks induced by Vickers indentation at 29.4 N load. (**a**) Upper layer, (**b**) middle layer, (**c**) lower layer. Shallow lateral cracks (L_1_, L_2_, and L_3_) located in the upper layer near the surface. The middle layer indicates the circular microcrack zone at the center and radial cracks outside the zone. There was only one median crack in the lower layer below the microcrack zone.

**Figure 4:**
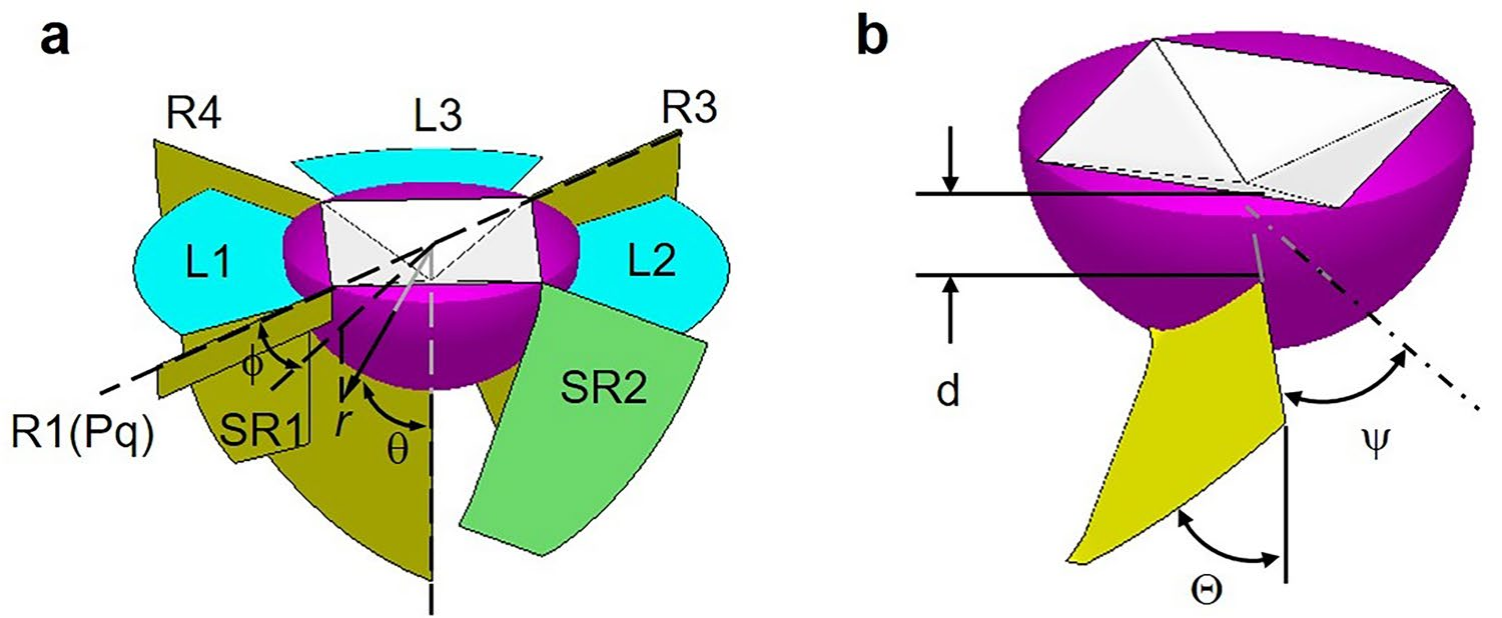
Schematic illustration of crack system induced by Vickers indentation around the indent and the semispherical process zone. (**a**) A crack model for loading at 29.4 N. Radial crack (R), secondary radial crack (SR), Palmqvist crack (Pq), lateral crack (L). Coordination system used to define the stress field at an indentation contact is illustrated. (**b**) Geometry of an inclined crack.

The subsurface crack system was divided into three layers in Fig. 3; (a) upper layer, (b) middle layer, and (c) lower layer. The shallow lateral cracks located in the upper layer near the surface (Fig. 3a). The topography of the surface of shallow lateral crack was rough and wavy, as shown in Supplementary Fig. S3 in detail. The rough surface is the origin of the brilliant appearance of lateral cracks of CAS-GC observed by optical microscopy. The rough crack surface has been attributed to crack deflection by plate-like CAS crystals, which form a house-of-cards structure^36^. The evidence of crack deflection was seen not only for lateral cracks, but also for median crack from the crack profiles presented in Fig. 2c. The middle layer (Fig. 3b) indicates the circular microcrack zone at the center and radial cracks outside the zone. In the lower layer below the microcrack zone (Fig. 3c), there was only one median crack, which was connected with radial crack R4. The connection between the median crack and radial cracks R2 and SR2 was not detected unambiguously. There was no median crack, which connected radial cracks R1 and R3. As Cook and Pharr^5^ pointed out, cracks emanating from the indentation corners are not always originated from the median cracks. Figure 4a illustrates the schematic representation of crack system by Vickers indentation around the indent and the semi spherical process zone.

### Comparison of micro- and nano-CT observations of Vickers indentation cracks at 9.8 N load

**Figure 5:**
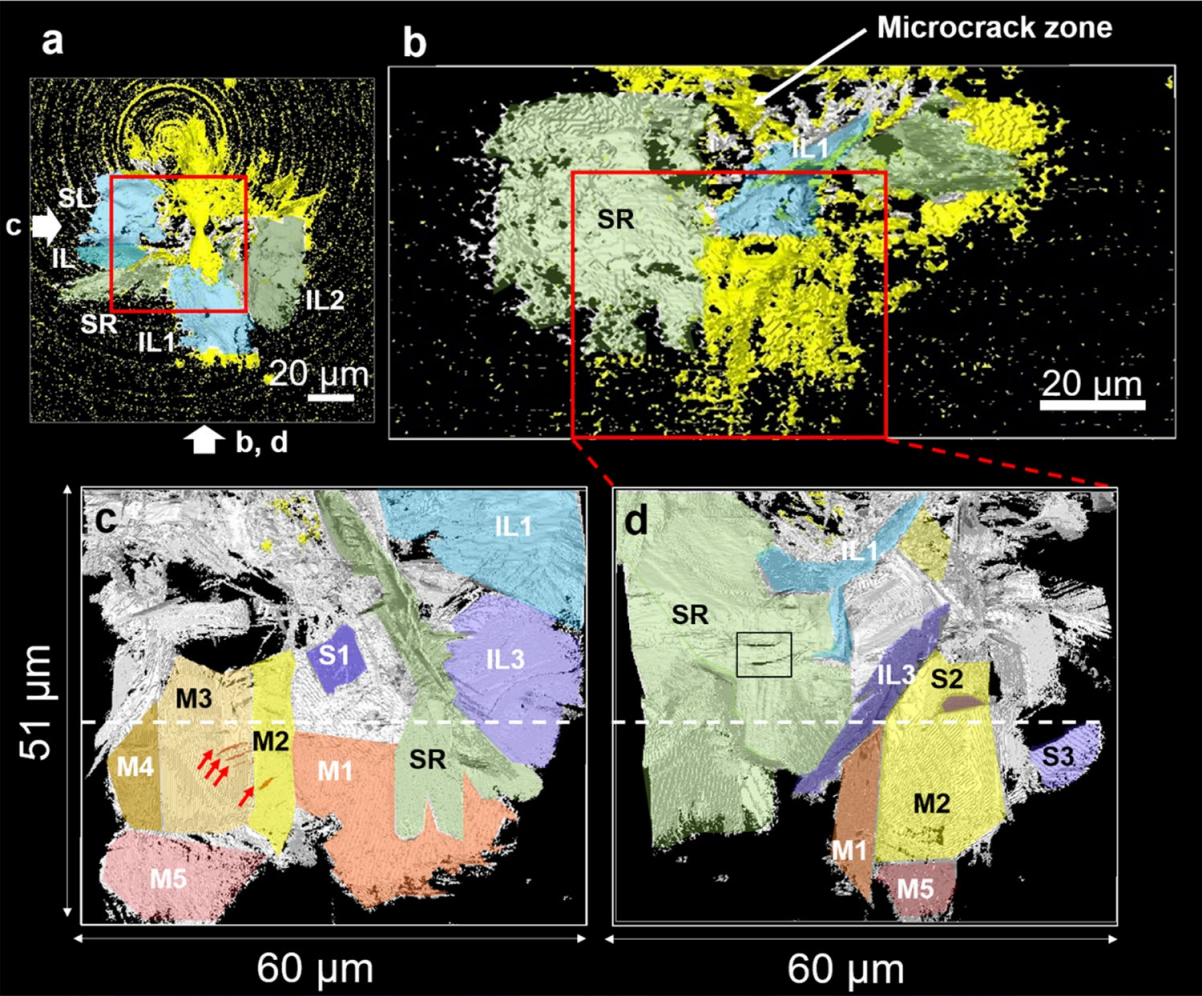
Micro-CT and nano-CT observation of Vickers indentation cracks at 9.8 N load. (**a**) Top view and (**b**) side view of micro-CT image. (**c**) and (**d**) side view of a region-of-interest [ROI, a cube shown as squares in (**a**) and (**b**)] observed by nano-CT. Arrows in (**a**) indicate the relative orientation of panels (**b**), (**c**), and (**d**). (SL: Shallow lateral crack, SR: Secondary radial crack, IL: Inclined lateral crack, M: Median crack, S: Single rectangular crack).

**Figure 6:**
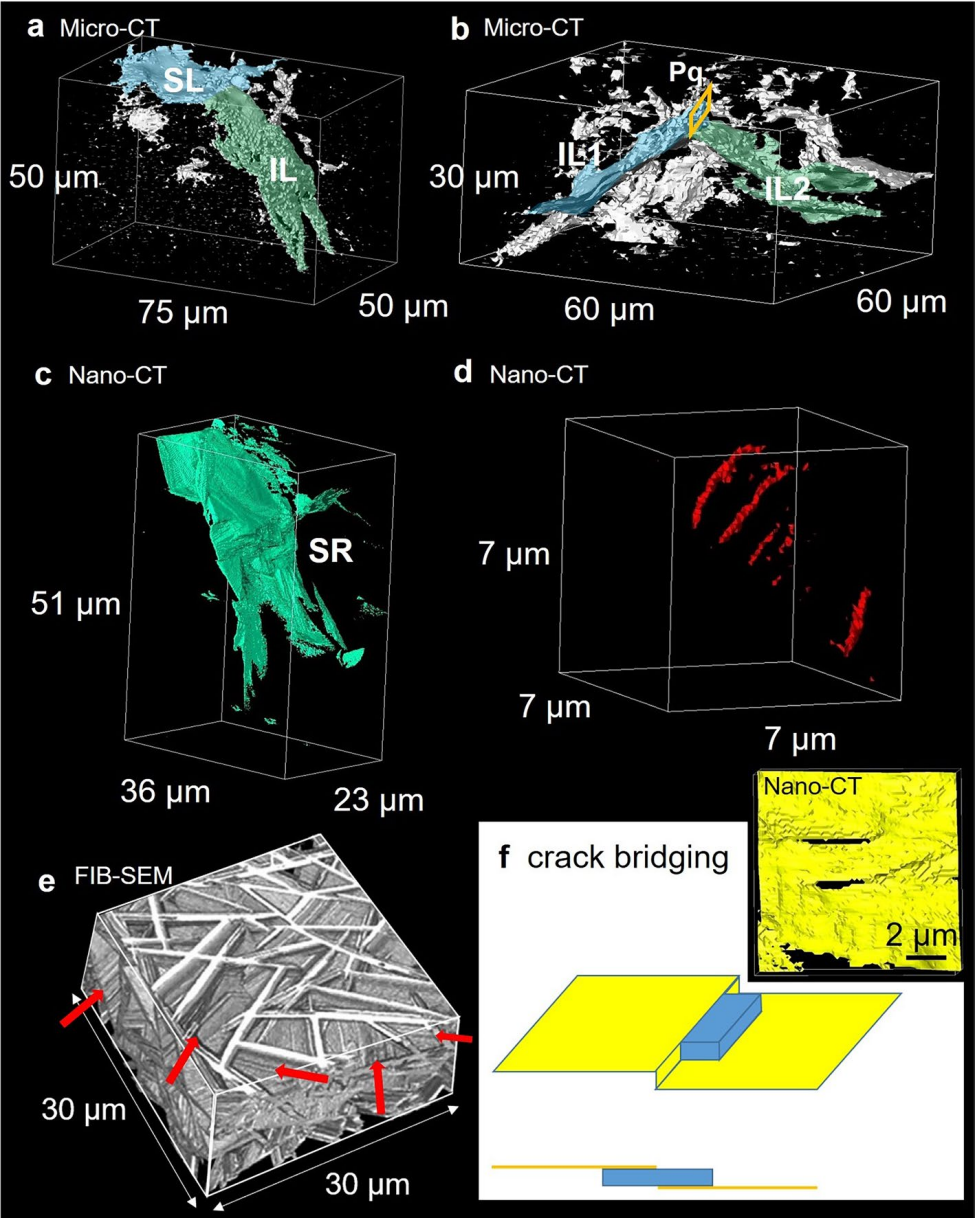
Components of indentation crack system. (**a**) Combined crack consisting of a shallow lateral crack (SL) and an inclined lateral crack (IL) in Fig. 5a, (**b**) combined crack consisting of a surface localized radial crack (Palmqvist crack, Pq) and two inclined lateral cracks (IL1, IL2) in Fig. 5a, (**c**) secondary radial crack (SR) in Fig. 5a,b, (**d**) parallel ribbon-like cracks in Fig. 5c (red arrows), (**e**) the house-of-cards structure of h-CAS crystals observed by FIB-SEM tomography, (**f**) two rectangular holes on secondary radial crack (SR) in Fig. 5d and a crack bridging model of a platelet located parallel to crack surface.

The indentation crack patterns become irregular under low loading, because the crack nucleation is affected by the microstructural heterogeneity. We observed carefully the complicated crack structure at 9.8 N load by using micro- and nano-CT [see Supplementary Movies 2 (micro-CT) and 3 (nano-CT)]. The top view and the side view of micro-CT image of the cracks are shown in Fig. 5a,b, respectively. They reveal that a diversity of cracks with different types, locations, and orientations, are formed outside the microcrack zone. Some complicated crack systems, (SL + IL) and (IL1 + IL2) in Fig. 5a observed by micro-CT, are considered as combined cracks consisting of several components as illustrated in Fig. 6a,b, respectively. The inclined lateral crack was identified as a new type of crack component. The inclined lateral crack (IL) initiates along a shallow lateral crack (SL) (Fig. 6a) or a surface-localized radial crack (Palmqvist crack, Pq) (Fig. 6b). The orientation of inclined lateral crack was different from that of radial crack and median crack (parallel to the load axis) or lateral crack (perpendicular to the load axis). While a shallow lateral crack^5^ propagates almost parallel to the surface, the inclined lateral crack goes into surrounding material at an angle to the load axis in a similar way to the secondary radial crack (SR2 in Fig. 4a). The orientation of the inclined lateral crack suggests a mode II crack driven by shear stress. Yoffe^4^ proposed that the stress field around the elastic–plastic deformation zone was given as the superposition of a point-contact field (Boussinesq field) and a blister field due to the permanent deformation beneath the indenter. The coordinate system used to describe the stress field is illustrated in Fig. 4a. Both Boussinesq field and blister field have the maximum shear stress $\tau_{r\theta }$ at $\theta = \pi /4$, then, this shear stress may affect the initiation and propagation of a mode II crack in the middle layer between the surface and the bottom of the process zone. The geometry of an inclined crack is defined by the initiation location (the depth *d* from the surface and the angle $\psi$) and the orientation ${\Theta }$ of the crack as shown in Fig. 4b; for example, $d > 0$, $\pi /2 >\Theta > \pi /4$ for inclined lateral crack, $d = 0$, $\psi > 0,$ $\pi /4 >\Theta > 0$ for secondary radial crack, $d = 0$, $\psi = 0,$ ${\Theta } = 0$ for radial crack, $d > 0$, $\Theta = \pi /2$ for lateral crack, and $d > R$, $\Theta = 0$ for median crack. Xie et al.^14^ and Baggott et al.^16^ reported lateral cracks interconnecting with radial crack beneath Vickers indentation on silicon nitride. Such crack structure can be also regarded as a combined crack system consisting of a radial crack and two inclined lateral cracks.

Micro-CT in Fig. 5b could detect only a part of crack surface with large crack opening displacement (COD), so that it gave the appearance of a collection of porous fragments. High resolution nano-CT observation was conducted to investigate the lower part of the microcrack zone and cracks formed outside the zone boundary (the region inside red squares in Fig. 5a,b). The side view of the region observed by nano-CT revealed the detailed crack structure. The inclined lateral crack (IL1) in Fig. 5c,d is a component of combined crack in Fig. 6b, which initiated below a Palmqvist crack ($R > d > 0$). The topography of the inclined lateral crack was rough and wavy in a similar way to the shallow lateral crack (Supplementary Fig. S3). An inclined lateral crack (IL3 in Fig. 5d) was also initiated in a region below the microcrack zone ($d > R$). On the other hand, a significant crack deflection and crack branching were observed in the secondary radial crack (SR) in Figs. 5c,d and 6c. There were several rectangular cracks (S1, S2, and S3). They looked like cracks at the interface between a single plate-like CAS crystal and glass matrix or cleavage plane, which initiated independently. The red arrows in Fig. 5c indicate a series of parallel cracks like ribbon strips. The enlarged view is illustrated in Fig. 6d. This crack structure is also related to a single rectangular h-CAS crystal. The house-of-cards structure was observed by FIB-SEM tomography^37^, and presented in Fig. 6e. The surface of platelet was not flat perfectly, but there were surface undulations due to the instability during crystal growth. The parallel cracks are localized microcracks at the interface generated by stress concentrations along the surface undulations.

**Figure 7:**
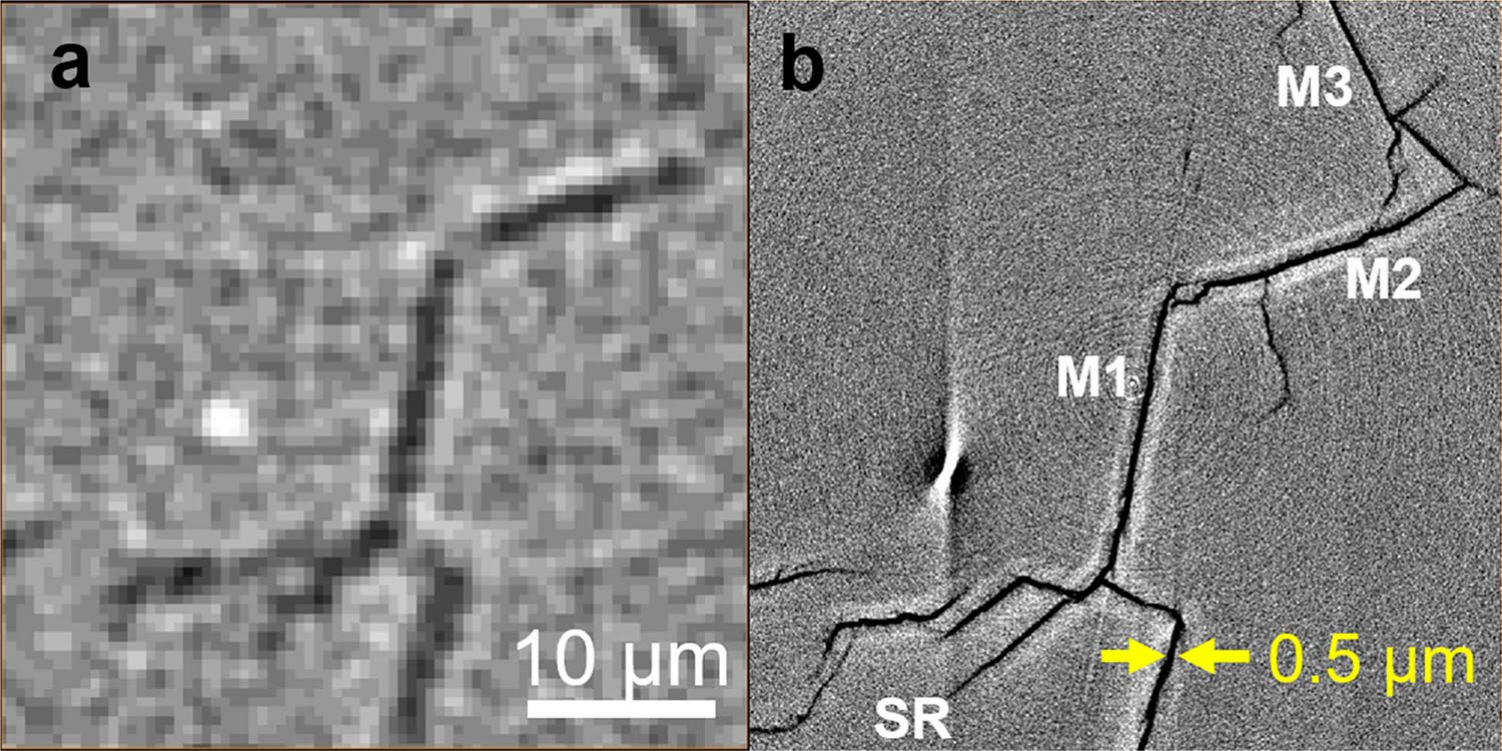
Median cracks. (**a**) Cross sectional image of median cracks observed by micro-CT, (**b**) Cross sectional image of median cracks observed by nano-CT.

The median cracks (M1–M5) were formed in the region below the microcrack zone (Fig. 5c,d). They are flat and parallel to the load axis. This is the region where two median cracks should be nucleated usually bisecting each other at right angles in the ideal model. However, the observed crack was a connected series of flat cracks. It suggested that multiple nucleation of median cracks occurred due to the house-of-cards structure in CAS-GC. The structure of median crack system was observed in the cross-section perpendicular to the loading axis at the position indicated by white dashed line in Fig. 5c,d. Although median cracks could be detected by micro-CT (Fig. 7a), the apparent COD was about 1.5–2 µm as the spatial resolution is typically 2–3 times the voxel size (0.5 µm). Nano-CT observation indicated that the actual size of COD was 0.5 µm (Fig. 7b).

## Discussion

The high fracture toughness of the CAS-GC (*K*_*IC*_ = 2.22 MPa m^1/2^ by SEVNB method) is attributed to crack deflection mechanism^28,29^. The fracture surface of CAS-GC after bending test was rough^34,36^ and consisted of flat facets indicating crack propagation along the interface between h-CAS crystals and glass or cleavage plane, because h-CAS crystal is analogous to mica^37,38^. The rough fracture surface was correlated with the house-of-cards structure^34,36^. The crack deflection of the secondary radial crack (SR1 in Figs. 5d, 6c) agreed with previous studies. On the other hand, the wavy profiles of lateral crack and inclined lateral crack (IL1 in Fig. 5c) indicate that the crack can propagates within the glass matrix and penetrate into h-CAS crystals, in addition to crack deflection^28,29^. Furthermore, the median cracks were quite flat. The observed diversity in crack profile suggests that the crack deflection depends on the crack type, the fracture mode, and the local stress field.

Two rectangular holes were observed on the surface of secondary radial crack (SR) in Fig. 5d (inside a rectangle). The holes locate on a crack surface with terrace-and-step morphology (Fig. 6f). This structure is related to the presence of rectangular h-CAS crystal on the crack surface as schematically illustrated in Fig. 6f. The upper part of the crack surface is partly bonded to the lower part via the h-CAS crystal. The toughening theory on crack bridging usually assumes reinforcements, fiber^30,31^ or platelets^40^, vertically located on the crack surface. The present observation showed that platelets parallel to the crack surface can contribute to the toughening by crack bridging.

**Figure 8:**
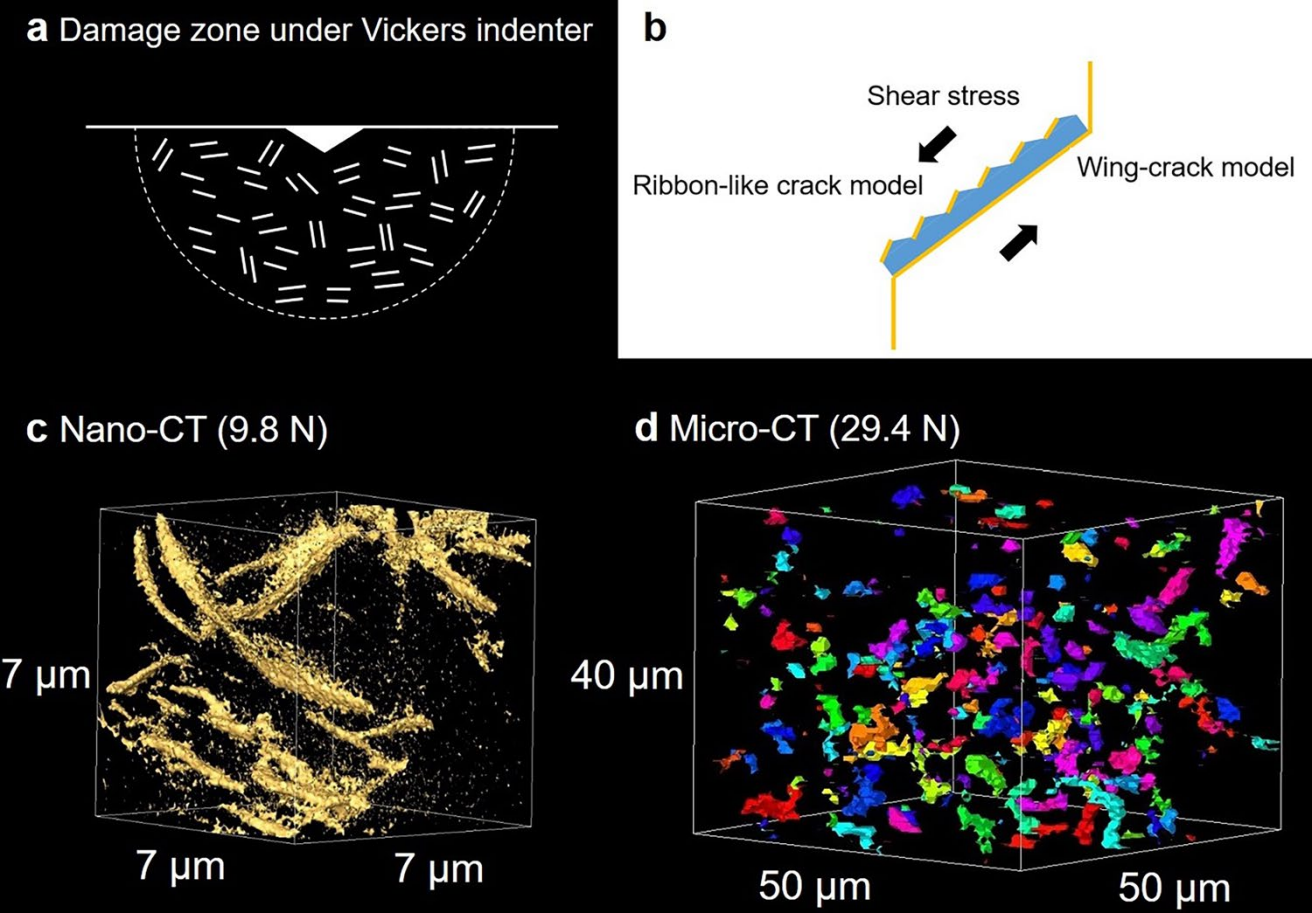
Ribbon-like crack/wing-crack models in the microcrack zone. (**a**) Damage zone under Vickers indenter, (**b**) formation of ribbon-like cracks on the modulated surface and that of wing-cracks on both sides of a flat surface of an individual plate-like crystal, (**c**) nano-CT image of the microcrack zone induced by Vickers indentation at 9.8 N, (**d**) micro-CT image of the microcrack zone induced by Vickers indentation at 29.4 N. Some rod-like defects are parallel.

The formation of microcrack zone by Vickers indentation was imaged by using micro-CT in CAS-GC with heterogeneous microstructure. Lawn^8^ reviewed that such damage zone, or quasi-plastic zone had been induced also in glass-ceramics and ceramics with heterogeneous microstructures beneath spherical indenters, in the region where the shear stress was maximum. The boundary of damage zone was determined by the shear stress contour. In the subsurface damage zone of mica glass-ceramics, shear-fault micro-failures (“closed” mode II cracks) occurred at weak interfaces between mica platelets and glass phase^9^. Lawn presented a model that the formation and the sliding at the shear fault would produce “wing” cracks on both sides of the shear fault at high loads. In CAS-GC, the shear stress would cause a fault along h-CAS crystal in the microcrack zone. The shear-fault/wing-crack system may be created at high loads as schematically illustrated in Fig. 8a,b. The crack opening displacement of the wing crack will be the maximum along the edge of h-CAS crystal. However, such wing-crack system could not be observed by nano-CT in microcrack zone formed under the load of 9.8 N (Fig. 8c). There were several ribbon-like cracks which resemble the localized microcracks at the interface of h-CAS crystal (Fig. 6d). As the surface of h-CAS crystal is modulated, ribbon-like cracks will be formed by shear stress (Fig. 8b). The micro-CT image of the microcrack zone induced by Vickers indentation at load of 29.4 N revealed the presence of rod-like defects, some of which were parallel (Fig. 8d). It is supposed that parallel ribbon-like cracks on the interface of platelets are observed as rod-like defects due to the resolution limit of micro-CT.

Fu and Evans^32,33^ analyzed the formation of microcrack around a primary crack, and proposed microcrack toughening. The microcracking reduces the elastic modulus of microcrack process zone, thereby, mitigating the stress field around the primary crack. Microcracking in heterogeneous glass-ceramics is assisted by the internal residual stress^27^, which arise during cooling due to the difference in thermal expansion and elastic constants. However, we could not detect such microcrack process zone around the median cracks by using nano-CT, although there were a few microcracks. The high fracture toughness of CAS-GC cannot be explained by microcrack toughening but by crack deflection and crack bowing.

The indentation crack pattern becomes symmetric with increasing load, because cracks can initiate at the maximum principal stress as the number of available crack nucleation site increases. The crack pattern is irregular at low load, since the crack initiation depends on the heterogeneous distribution of CAS crystals as the available nucleation sites decrease. Therefore, new type of inclined crack (Fig. 4b) was formed when the crack nucleated at irregular site. Supplementary Fig. S4 indicates the early stage of crack initiation at very small load (400 mN)^37^. The cracks initiate along the cleavage plane of CAS crystal or the interface between crystal phase and glass stochastically.

In summary, we investigated the complex morphology of subsurface cracking induced by Vickers indentation in a glass ceramic with heterogeneous microstructure by using synchrotron X-ray multiscale tomography. The details of crack shape could be imaged by nano-CT, while the overall structure of complex crack system was observed by micro-CT. The complicated crack morphology arose due to the stochastic nature of crack nucleation in the inhomogeneous microstructure. Some crack systems were expressed as combined crack consisting of several components. The knowledge of subsurface crack morphology, which can never be observed on the surface, are required as a sound basis for characterizing the mechanical behaviors by indentation technique^41,42^. The multiscale tomography provides an opportunity to investigate the diversity of subsurface crack morphology in toughened materials with heterogeneous microstructures, glass ceramic, ceramics, hard metals and composites, systematically.

## Methods

### Materials

The precursor glass composition of 25CaO–20Al_2_O_3_–55SiO_2_ wt% was selected considering the low-liquidus temperature with an adequate viscosity. The precursor glass was prepared from related raw materials by melting at 1550 °C. For promoting crystallization, small amount of MoO_3_ was added into the mixture of the raw materials. MoO_3_ was reduced to metallic state during melting or heat treatment process by co-added reducing agents, and served as a nucleating agent of the hexagonal CaAl_2_Si_2_O_8_ crystals^43^. For the sample of X-ray tomography observation, 0.05 wt% of MoO_3_ and 0.4 wt% of carbon powder were added, whereas 0.005 wt% of MoO_3_, 0.4 wt% of carbon powder, and 0.28 wt% of TiO_2_ were added in case of the sample for optical microscopy observation shown in Fig. 1. The glass sample was heat treated at 1050 °C for 2 h for crystallization^32^. This glass ceramic (CAS-GC) contained ~ 21 wt% hexagonal CaAl_2_Si_2_O_8_ crystals with sizes less than 10–20 µm and the thickness of ~ 1 µm. The plate-like crystals formed a house-of-cards structure^35^. For X-ray tomography, cylindrical samples with 0.85 mm in diameter and 7 mm in length were fabricated from the bulk CAS-GC. Vickers indentation was carried out on the polished top surface of samples which were bundled by resin as illustrated in Supplementary Fig. S5. The indentation was performed using Vickers hardness tester (Matsuzawa Via, Akita, Japan) at loads of 9.8 N and 29.4 N. These loads were selected, because the cylindrical sample was broken at 49 N load.

### X-ray tomography

The 3D structure of subsurface cracks was studied by using the synchrotron X-ray multiscale-CT consisting of a micro-CT (microtomography) as a wide-field and low-resolution system and a nano-CT (nanotomography) as a narrow-field and high-resolution system at BL20XU of the Japanese synchrotron radiation facility, SPring-8^19,20^. X-ray energy was set at 20 keV for micro- and nano-CT mode. Optical system of nano-CT mode was based on a phase contrast X-ray full-field microscope. A hollow-cone illumination system using a condenser zone plate (CZP), sample stages, a Fresnel zone plate (FZP) objective, and a Zernike phase plate (phase ring) were placed at the experimental hutch 1. A visible-light conversion type X-ray image detector (C12849-SY69701, Hamamatsu Photonics, Shizuoka, Japan) was installed at the 2nd hutch located ~ 160 m downstream from the 1st hutch. The sample was rotated by steps of 0.1° up to 180°. Voxel sizes for micro- and nano-CT mode were 0.5 μm and 40 nm, respectively. The measuring time for one sample was ∼8 min and ∼30 min for micro- and nano-CT, respectively. The micro-CT is used to capture the entire structure of indentation cracks, and its region-of-interest (ROI) is measured with nano-CT precisely.

### Image analysis

The images were reconstructed from the acquired data by using a filtered back-projection method. Gaussian filtering was applied to reduce the noise in 2D images. The 3D visualization and geometrical measurements were performed using Amira (VSG, Burlington, Massachusetts, USA) and Dragonfly (Object Research Systems (ORS) Inc.). Local thresholding method was used to segment the gray value image into crack and material. The surface was discretized using triangular meshing.

## Supplementary Information

Supplementary Video 1.

Supplementary Video 2.

Supplementary Video 3.

Supplementary Information 1.

## Data Availability

The datasets used and/or analyzed during the current study available from the corresponding author on reasonable request.
