# Supplementary material for: Morphology of subsurface cracks in glass-ceramics induced by Vickers indentation observed by synchrotron X-ray multiscale tomography
Source: Sci Rep. 2022 Apr 28;12:6994. doi: 10.1038/s41598-022-11084-0 (PMC9050884; doi:10.1038/s41598-022-11084-0)
Supplement: Supplementary file 4 — Supplementary Information 1. [file 41598_2022_11084_MOESM4_ESM.pdf]

Supplementary Information

Morphology of subsurface cracks induced by Vickers indentation observed by synchrotron X-ray multiscale tomography: a case study of $CaO-Al_2O_3-SiO_2$ glass-ceramic

Gaku Okuma[1*], Kei Maeda[2], Satoshi Yoshida[3], Akihisa Takeuchi[4], Fumihiro Wakai[1]

[1] Research Center for Structural Materials, National Institute for Materials Science, 1-2-1 Sengen, Tsukuba, Ibaraki 305-0047, Japan

[2] Department of Materials Science and Technology, Tokyo University of Science, 6-3-1 Niijuku, Katsushika-Ku, Tokyo 125-8585, Japan

[3] AGC Inc. Yokohama Technical Center, 1-1 Suehiro-cho, Tsurumi-ku, Yokohama 230-0045, Japan

[4] Japan Synchrotron Radiation Research Institute, JASRI/SPring-8, Kouto 1-1-1, Sayo, Hyogo, 679-5198, Japan

*Corresponding author: OKUMA.Gaku@nims.go.jp

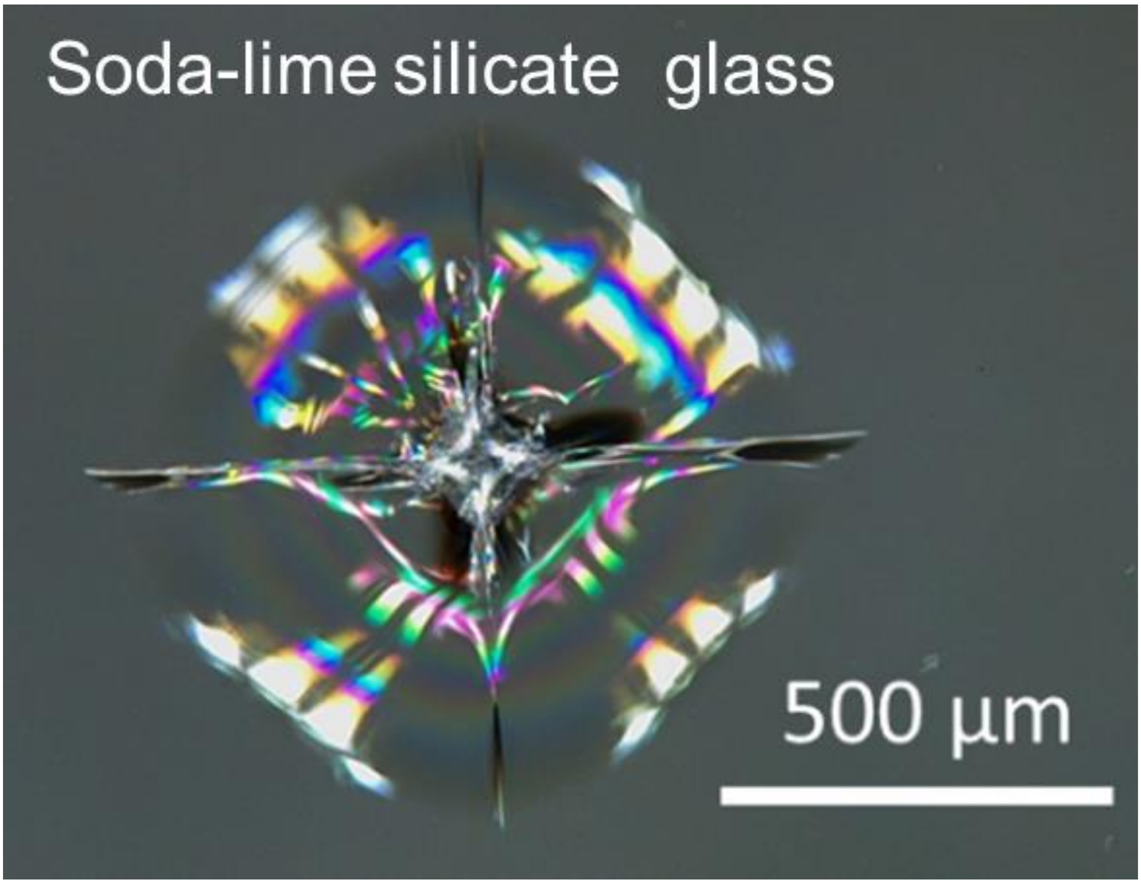

Supplementary Figure S1. Optical micrograph of cracks around the imprint of Vickers indentation at 98 N in soda-lime silica glass (SLS)[34]. The observed lateral cracks are not brilliant.

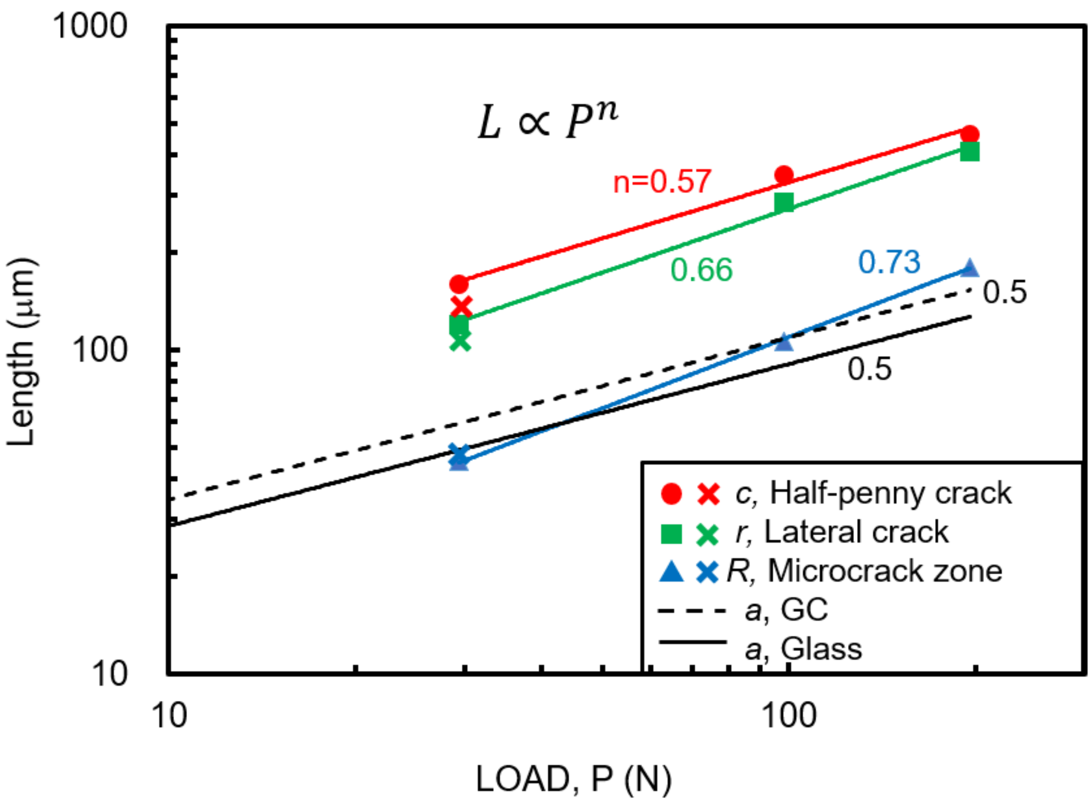

Supplementary Figure S2. Crack lengths (*c*, *r*, *R*) and indentation length (*a*) as functions of load *P* in Vickers indentation of CAS-GC measured by optical microscope. The indentation lengths of the original glass and CAS-GC were calculated from their hardness. ×-marks show the values measured by micro-CT at 29.4 N.

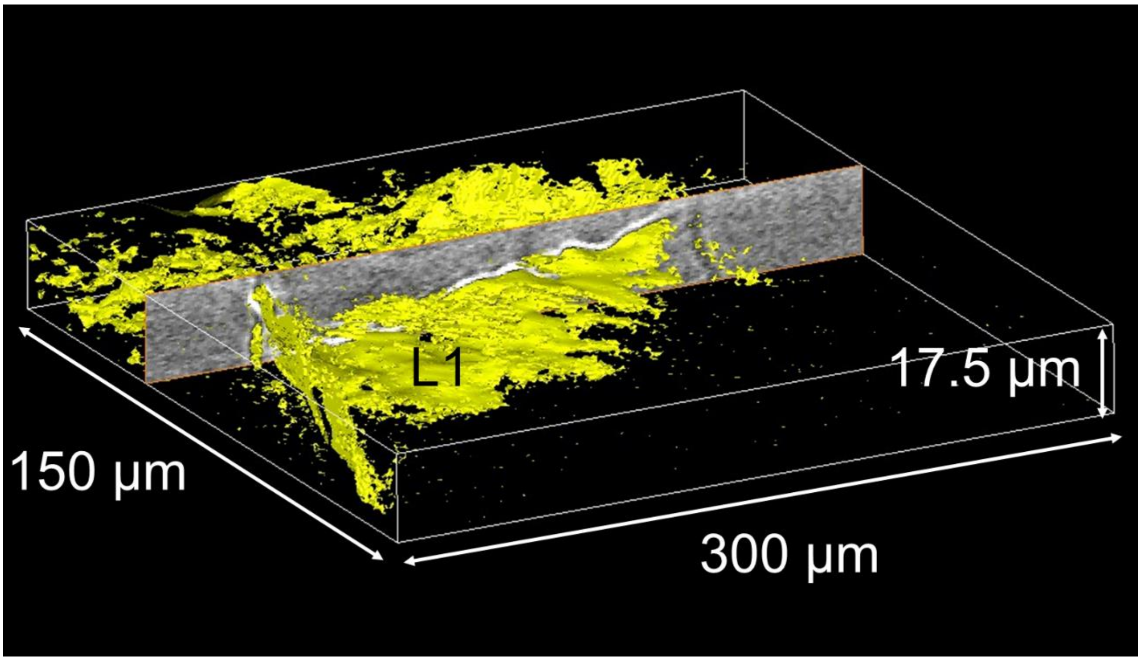

Supplementary Figure S3. Micro-CT image of lateral crack (L1) induced by Vickers indentation at 24.9 N.

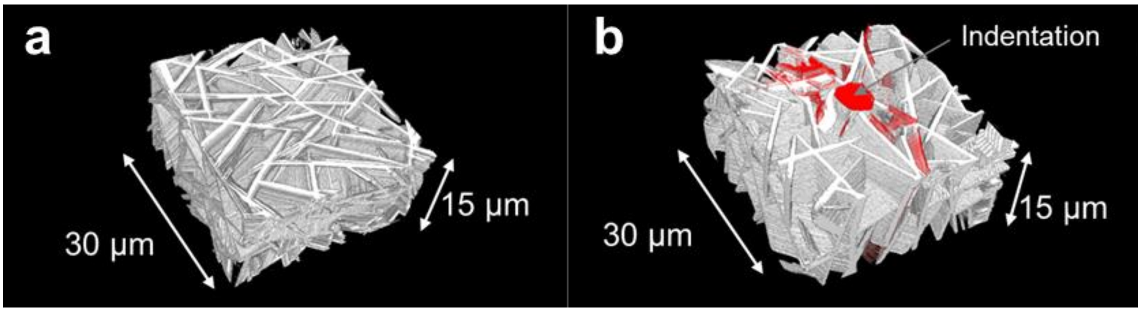

Supplementary Figure S4. (a) FIB-SEM tomography image of a three-dimensional (3D) microstructure in which the plate-like crystals with a thickness of ~1 µm formed a house-of-cards structure. (b) Microcracks induced by sharp indenter with small load (400 mN) along the cleavage plane or the interface between crystal phase and glass phase[36].

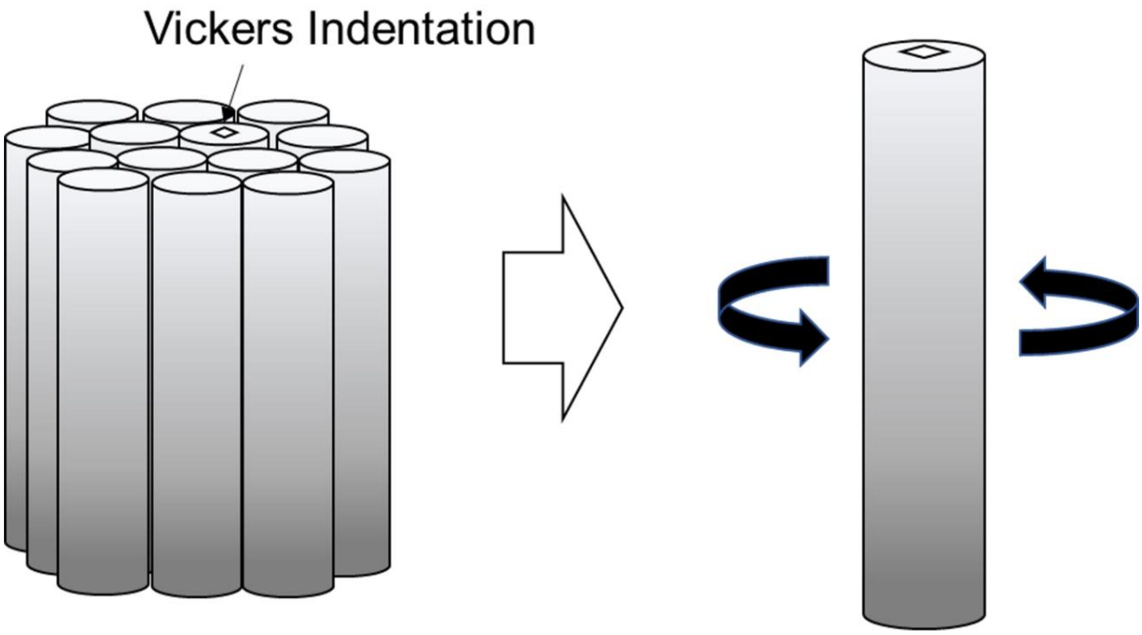

Supplementary Figure S5. Schematic illustration of the sample with Vickers indentation for X-ray multiscale-CT.
